# Coronary Artery Anomalies in Animals

**DOI:** 10.3390/vetsci4020020

**Published:** 2017-04-12

**Authors:** Brian A. Scansen

**Affiliations:** Department of Clinical Sciences, Colorado State University, Fort Collins, CO 80523, USA; Brian.Scansen@colostate.edu; Tel.: +1-970-297-5032

**Keywords:** heart, congenital, single coronary, veterinary, dog, cat

## Abstract

Coronary artery anomalies represent a disease spectrum from incidental to life-threatening. Anomalies of coronary artery origin and course are well-recognized in human medicine, but have received limited attention in veterinary medicine. Coronary artery anomalies are best described in the dog, hamster, and cow though reports also exist in the horse and pig. The most well-known anomaly in veterinary medicine is anomalous coronary artery origin with a prepulmonary course in dogs, which limits treatment of pulmonary valve stenosis. A categorization scheme for coronary artery anomalies in animals is suggested, dividing these anomalies into those of major or minor clinical significance. A review of coronary artery development, anatomy, and reported anomalies in domesticated species is provided and four novel canine examples of anomalous coronary artery origin are described: an English bulldog with single left coronary ostium and a retroaortic right coronary artery; an English bulldog with single right coronary ostium and transseptal left coronary artery; an English bulldog with single right coronary ostium and absent left coronary artery with a prepulmonary paraconal interventricular branch and an interarterial circumflex branch; and a mixed-breed dog with tetralogy of Fallot and anomalous origin of all coronary branches from the brachiocephalic trunk. Coronary arterial fistulae are also described including a coronary cameral fistula in a llama cria and an English bulldog with coronary artery aneurysm and anomalous shunting vessels from the right coronary artery to the pulmonary trunk. These examples are provided with the intent to raise awareness and improve understanding of such defects.

## 1. Introduction

Coronary arteries perfuse the heart and facilitate nutrient and oxygen delivery to the metabolically active myocardium. Myocardial ischemia related to atherosclerosis and coronary artery obstruction is currently the leading cause of human death and is projected to remain so beyond the year 2030 [1]. Myocardial ischemia and infarction related to coronary artery disease also occur in dogs and cats [2,3], but are more often associated with other underlying diseases and are not believed to commonly impact the morbidity and mortality of domesticated species.

Congenital anomalies of coronary artery anatomy in man are important as a cause of non-arteriosclerotic ischemia and present an increased risk of sudden death in young athletes [4,5]. Coronary artery anomalies (CAA) in dogs are rarely reported to be of clinical significance, unless in the setting of pulmonary valve stenosis (PS) when the coronary anatomy limits intervention [6,7]. The aim of this manuscript is to review the coronary arterial circulation of animals, highlighting recognized anomalies and their clinical or comparative anatomical importance.

## 2. Coronary Artery Embryology and Anatomy

Connection of the coronary arteries to their appropriate aortic valvar sinuses occurs late in development, after closure of the interventricular septum and coincident with development of the aortic root and semilunar valves [8]. Early in development, the coronary arteries develop as layered epithelial cells filled with erythrocytes (called sinusoids or blood islands), which proliferate and conjoin to form a network of vascular channels [9]. As venous connections develop, these primitive vascular channels are drained to the coronary sinus. The coronary arteries are eventually connected to the aortic valvar sinuses, allowing antegrade flow from aorta through the coronary vascular bed [9]. The process by which the coronary arteries connect to the aorta is an area of uncertainty with recent reports based upon murine data describing endocardial strands growing from the aortic root as the outflow tracts are undergoing septation [8]. Previously, it was thought that the developing epicardial coronary arteries grew toward and then into the developing aortic root [9].

**Figure 1:**
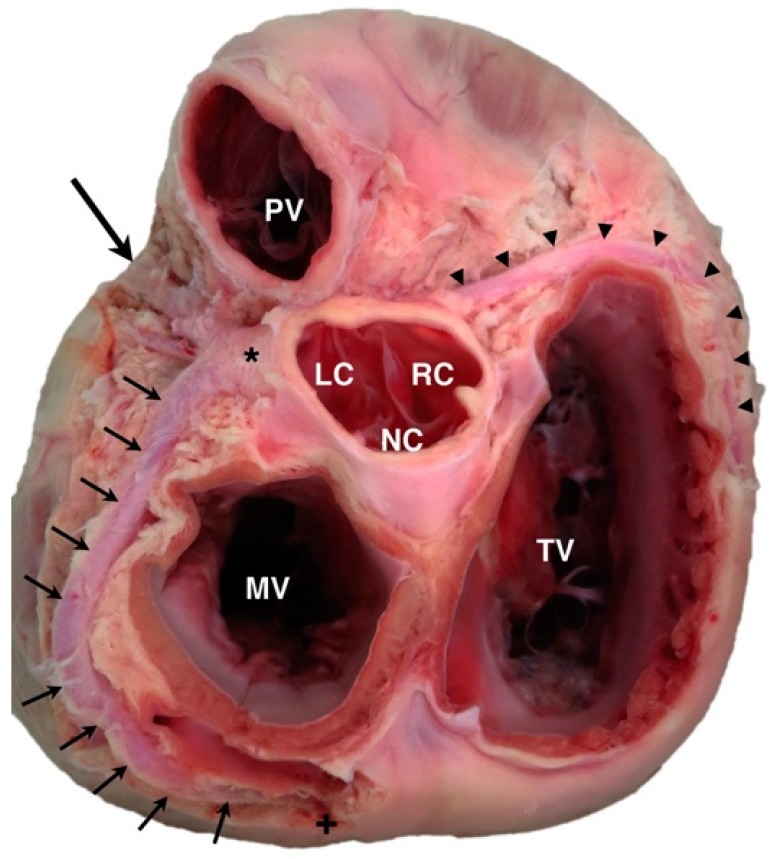
Canine specimen of the heart base, viewed from the dorsal perspective. The left coronary artery (*) can be seen arising from the left-adjacent cusp (LC) of the aortic valve and dividing into the paraconal interventricular branch (large arrow) and circumflex branch (multiple small arrows), which wraps around the mitral valve (MV) annulus to descend as the subsinuosal interventricular branch (+). The right coronary artery (arrowheads) can be seen arising from the right-adjacent cusp (RC) of the aortic valve and passing to the right and caudal of the tricuspid valve (TV) annulus. PV = pulmonary valve; NC = non-adjacent cusp of aortic valve.

**Figure 2:**
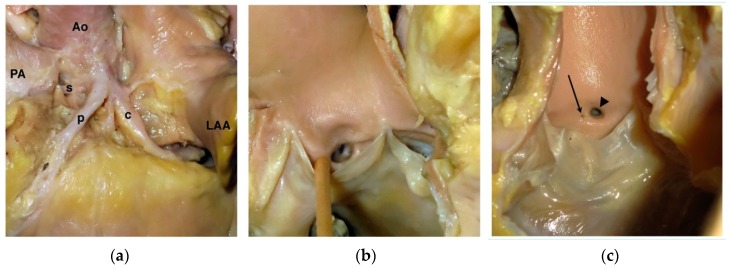
Variations of normal coronary ostia in the dog. (**a**) Left coronary trifurcation in a dog with a very short (nearly absent) left main coronary artery in whom the left coronary ostium gives rise to a septal branch (s), paraconal interventricular branch (p), and a circumflex branch (c). Ao = aorta; LAA = left auricular appendage; PA = pulmonary artery. (**b**) View of the left aortic valvar sinus from the dog in panel (a) showing the trifurcation of the left coronary system. (**c**) Photograph of the right aortic valvar sinus from a different dog showing a right coronary ostium (arrowhead) with an additional small ostium (arrow) consistent with the right accessory or conus branch.

Ostia (openings), vessels (individual arteries), and their directional course around and within the heart are all described when considering the coronary artery anatomy. In the normal mammalian heart, the aortic valve sinuses are adjacent (facing) the right and left aspects of the pulmonary valve while the third sinus is non-adjacent. To maintain appropriate anatomical terminology in a congenitally-malformed heart where the position of the great vessels may be altered, the aortic sinuses are therefore named as if they were viewed from the non-adjacent aortic sinus and facing the pulmonary valve giving a right aortic sinus or cusp, a left aortic sinus or cusp, and a non-adjacent sinus or cusp [8]. In the vast majority of animal hearts, the two coronary ostia are located within the left and right aortic valvar sinuses adjacent to the pulmonary trunk (Figure 1). Commonly, a third ostium is present in the right valvar sinus representing a small conus branch (Figure 2C), known as the right accessory coronary artery [10], that arborizes across the right ventricular outflow tract. Two ostia may also be present in the left valvar sinus representing separate origins of the paraconal interventricular (Pc) and circumflex (Cx) arteries [11] or a separate ostium for the septal branch distinct from the left coronary artery (LCA) [12]. The coronary ostia are typically located in the middle of the valvar sinus and below the sinotubular junction (Figure 2). If they originate above the sinotubular junction, by at least 1 cm in a human, they are termed to have a high take-off.

The normal LCA arises from the left coronary ostium, comprises a short main segment, and then bifurcates into the Pc and Cx branches (Figure 1), or trifurcates into the Pc, Cx, and septal branches. In some dogs, there is no discrete LCA and the left coronary branches arise directly from the left coronary ostium (Figure 2A,B). The Pc branch descends obliquely on the left face of the heart and to the ventricular apex within the paraconal interventricular groove. The Cx branch runs within the coronary groove along the atrioventricular junction and encircles the mitral valve annulus. The septal branch differs in the dog as compared to the human or equine anatomy because it provides the majority of the blood supply to the interventricular septum and may in rare cases be larger than the Pc branch [12,13]. In the dog, the septal branch arises either off the LCA, directly from the left coronary ostium, or off the Pc branch and descends adjacent to the pulmonary valve annulus lying within the subendocardium along the right ventricular aspect of the septum. The subsinuosal interventricular branch arises variably from the LCA or right coronary artery (RCA), predominately related to species differences discussed below; it descends along the caudal border of the heart within the subsinuosal interventricular groove. The RCA arises from the right coronary ostium and courses to the right and caudally within the right atrioventricular groove to supply the right ventricular mass (Figure 1); as noted above, a small accessory branch often is seen extending cranially from the RCA or directly from the right aortic valvar sinus toward the right ventricular outflow tract. However, in the normal animal heart, the major CAs are not found in a position cranial to the right ventricular outflow tract or pulmonary valve annulus.

The coronary dominance of a heart refers to the coronary artery (LCA or RCA) that perfuses the majority of the myocardial tissue and variable methods to determine this have been reported including which coronary artery supplies the subsinuosal interventricular branch, which coronary artery extends beyond the crux of the heart, the relative lengths and number of branches of the LCA or RCA, and the origination and layout of the arteries at the apex of the heart [14]. The dog normally displays a left dominant coronary circulation [14], the cat is less studied but appears variable with a majority of cases indicating right coronary dominance [15], the rabbit is variable with predominately left coronary dominance [16], and the goat [17], swine [18], horse and donkey [19], cow, camelids [20], and 90% of humans [21] are right dominant. In the mouse [22] and rabbit [23], the subsinuosal interventricular branch is reported to be absent. Notably, a report of 10 chinchillas found that all dissected specimens lacked an RCA suggesting that a single LCA may be normal in that species [24]. In both humans and veterinary species, balanced or co-dominant coronary circulations are observed in which both the LCA and RCA supply the diaphragmatic aspect of the myocardial mass. Species variability in coronary dominance has importance as experimental studies of coronary occlusion in the dog result in different regions of myocardial ischemia and unique collateral pathways than would occur in species with a right dominant coronary circulation.

## 3. Prevalence and Categorization of Coronary Artery Anomalies

**Figure 3:**
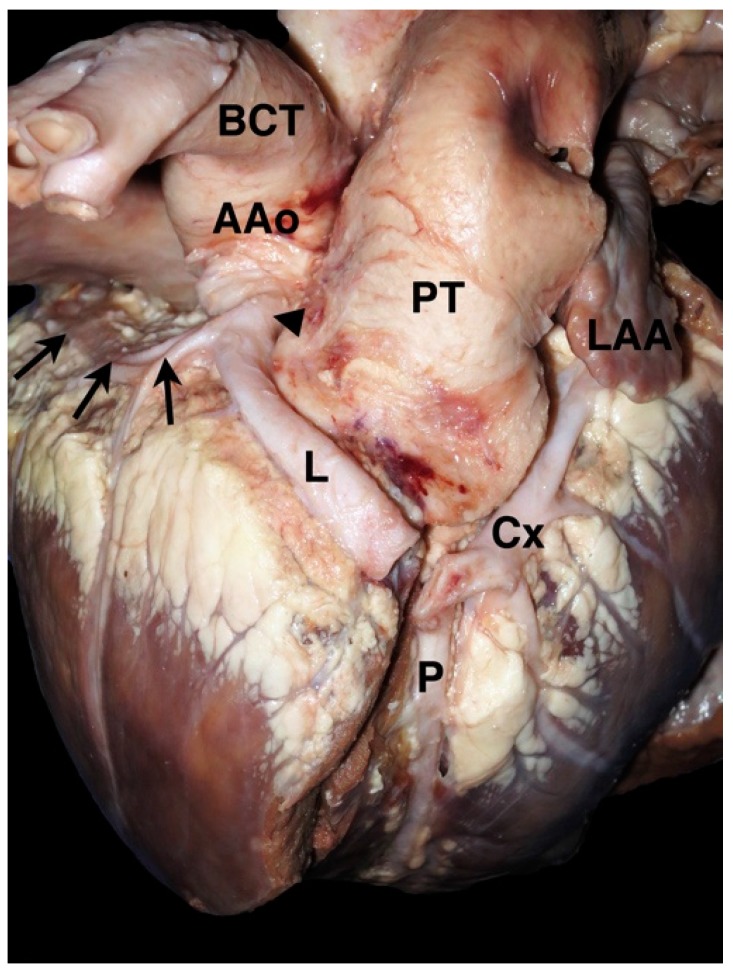
Photograph from a 2-month-old calf with double outlet right ventricle and a coronary artery anomaly. A single right coronary ostium (arrowhead) gives rise to the right coronary artery (arrows) as well as the left main coronary artery (L), which crosses cranial (prepulmonary) to the pulmonary trunk (PT) before dividing into the paraconal interventricular branch (P) and the circumflex branch (Cx). Note that the autopsy cut along the interventricular groove has transected the left coronary artery. AAo = ascending aorta, BCT = brachiocephalic trunk, LAA = left auricular appendage.

Compared to humans, reports of congenital CAA in animals are sparse, presumably because coronary artery disease is less common in veterinary species and therefore diagnostic coronary arteriograms are not routinely performed. The estimated prevalence of CAA in humans is difficult to quantify, due to a lack of agreement on what constitutes an anomaly versus a variant of normal [4]. The prevalence has been cited as high as 5.64% of humans, though many if not most of these defects were not clinically significant [4]. To clarify the uncertainties in prevalence, it has been suggested that only those variants that are present in less than 1% of the general population be considered as true anomalies of the coronary artery circulation [4]. Complications of CAA in humans relate to myocardial ischemia from ostial narrowing, extramural compression, or increased risk of atherosclerotic disease [4]. Coronary artery anomalies are seen with greater incidence in humans with congenital heart disease, particularly in conditions associated with malposition of the aortic root. Up to a third of patients with transposition of the great arteries and a quarter of patients with congenitally-corrected transposition of the great arteries have abnormal coronary artery anatomy; up to 14% of patients with tetralogy of Fallot have abnormal coronary artery branching with 5% of cases having a major coronary artery branch crossing anterior (cranial) to the pulmonary valve annulus; up to 18% of patients with common arterial trunk have a single coronary ostium; and a third or more of cases with double outlet right ventricle display abnormal coronary artery anatomy [25,26]. Studies of coronary artery anatomy from animals with congenital heart disease are not available, but the author’s impression, based on evaluation of numerous post mortem specimens, is that a similar proportion of animals, both large and small animal species, with complex congenital heart disease such as double outlet right ventricles and transposition complexes have CAA. Figure 3 shows an example of a cow with double outlet right ventricle and a CAA; in this animal, a single right coronary ostium provided the coronary arterial circulation with the LCA taking a prepulmonary course.

Classification schemes to report CAA are inconsistent in human medicine and even more variable in animal reports. Table 1 attempts to categorize major anomalies that are likely to be associated with clinical signs or cardiac complications as compared to those that are more likely to have minor clinical impact. In people, the major CAAs cause profound myocardial ischemia or high risk of congestive heart failure. Some of the minor anomalies, however, have also been implicated in sudden death, particularly during exertion in young athletes [4,27]. Sudden death appears most often when an anomalous coronary artery origin results in ostial stenosis, an acute angle as it exits the aortic wall, an intramural course (within the aortic wall), or an interarterial course between the great vessels [4,27]. The risk of sudden death with a minor CAA in animals has not been established. Descriptive naming of specific anatomy is now preferred to alphanumeric coding schemes [28].

### Veterinary Reports of Coronary Artery Anomalies

Dogs and cattle are the veterinary species most frequently reported with CAA, though reports also exist in the horse and pig. Reports of CAA in dogs include the single right coronary ostium with an anomalous prepulmonary LCA first reported in 1959 in a necropsy study of an asymptomatic mongrel dog [29] and later characterized in association with PS [6,7,30,31,32], single left coronary ostium with an anomalous prepulmonary RCA [33,34], single right coronary ostium with suspected interarterial LCA in an English bulldog [35], single right coronary ostium in a Keeshond with common arterial trunk [36], single right coronary ostium in a Collie with double outlet right ventricle [37], anomalous origin of the LCA from the pulmonary trunk in a miniature poodle [38], LCA aneurysm in a German shepherd dog with subaortic stenosis [39], coronary to pulmonary artery fistula in a German shepherd dog [40], and incidental myocardial bridging in many dogs found on autopsy [41,42]. In the cow, single right coronary ostium with an interarterial LCA [43], single right coronary ostium with a prepulmonary LCA and coronary-to-pulmonary artery fistula [9], LCA to left ventricle fistula [44], dual origin of the LCA [45], left circumflex branch to right ventricle fistula [46], and several cases of anomalous origin of the LCA from the pulmonary trunk [47,48,49] have been described. In the horse, single right coronary ostium with a prepulmonary LCA [50] as well as single right coronary ostium with an interarterial LCA have been reported [51]. In the pig, single left coronary ostium with an anomalous RCA has been reported as an incidental finding [52]. The Syrian hamster has been proposed as an animal model of CAA with numerous forms of anomalous coronary artery origin and course described from inbred research colonies [53,54], as well as LCA from the pulmonary trunk [55] and hypoplastic or rudimentary coronary arteries [56].

## 4. Anomalies of Coronary Origin and Course

### 4.1. Anomalous Origin from the Aorta

Coronary arteries that arise anomalously from the aorta have previously been categorized in human and veterinary literature using the angiographic classification of Lipton [58]. In this system, naming is based upon the vessel’s origin from the right (R) or left (L) aortic valvar sinus, whether only one (I), two (II), or three (III) branches of the major coronary arteries are present, and the course of the anomalous vessel, whether anterior (A), between (B), or posterior (P) to the great vessels [59]. This was the naming scheme adapted by Buchanan in his report [6] of the single right coronary ostium with an anomalous prepulmonary left coronary artery (so-called R-II-A anatomy) in four dogs. The description in the title of that report [6] (single coronary artery) differs from current nomenclature as those four dogs did not have single coronary artery anatomy—all coronary arteries were present, but the left was anomalous in origin (arising from the right coronary ostium) and course (prepulmonary). Single coronary artery anatomy (R-I or L-I by this classification) exists when there is only one major artery that terminates in both the right left branches [59] and has not been comprehensively described in an animal, to the author’s knowledge. Terminology of anomalous aortic origin, particularly the single coronary ostium that is observed in dogs, has evolved in human medicine with most recent articles referring to these as anomalous aortic origin of the coronary artery (AAOCA) or anomalous origin of a coronary artery from the opposite sinus (ACAOS) with modifiers to describe the artery involved (left or right) and the proximal course [27,60].

**Figure 4:**
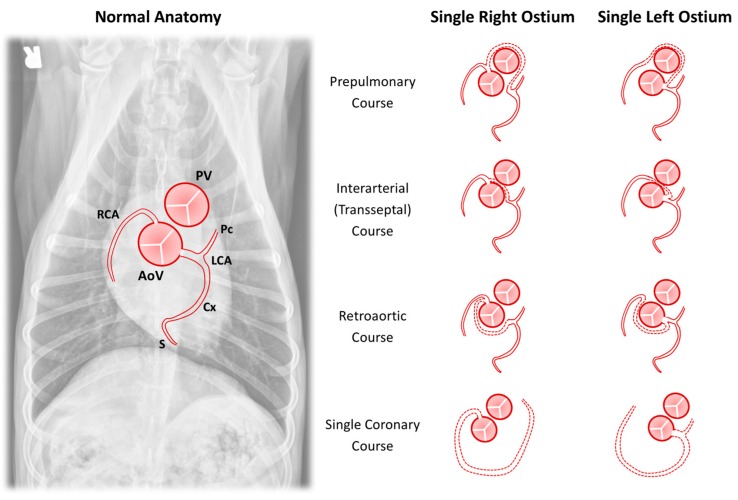
Schematic representation of anomalies of coronary arterial origin and course. The normal anatomy is shown superimposed on a ventrodorsal radiograph and this same orientation is maintained for each drawing. The drawings demonstrate variations in anomalous course (dashed lines) for the major coronary arterial branches in the setting of either a single right or single left coronary ostium. AoV = aortic valve; Cx = circumflex branch; LCA = left coronary artery; Pc = paraconal interventricular branch; PV = pulmonary valve; RCA = right coronary artery; and S = subsinuosal interventricular branch.

While the prepulmonary course of an anomalous coronary artery is considered only a minor anomaly in Table 1 because the anomalous vessel provides normal systemic blood supply to the myocardium, this anatomy in the dog has been associated with greater clinical significance in the setting of PS. The prepulmonary course of an anomalous coronary artery becomes significant if surgical or catheter-based therapies are planned, specifically right ventriculotomy or balloon pulmonary valvuloplasty, as damage to the coronary artery in this location may occur and can be fatal [31,61]. The association between PS and anomalous coronary artery anatomy was first reported by Buchanan and Patterson [62] in an English bulldog and further characterized in subsequent reports [6,30]. Additional reports in English bulldogs and boxer dogs have found alternative variants of the single coronary ostium including a single left coronary ostium with a prepulmonary RCA course [33] and a single right coronary ostium with a presumed interarterial LCA course [35]. As variants of this anatomy appear common in brachycephalic dog breeds, the differing arterial courses associated with a single coronary ostium are shown schematically in Figure 4.

The difference between the interarterial and transseptal course reflects the position of the anomalous course—with the interarterial course lying between the pulmonary trunk and aorta and the transseptal traversing lower (more apical) and typically surrounded by ventricular myocardium [28], though some authors dispute this distinction [63,64]. As in humans, it is likely that the anomaly of greatest clinical significance is an interarterial course due to risk of myocardial ischemia [4,43]. If pulmonary stenosis is concurrently diagnosed, the prepulmonary course is significant due to potential for damage during balloon valvuloplasty [61].

**Figure 5:**
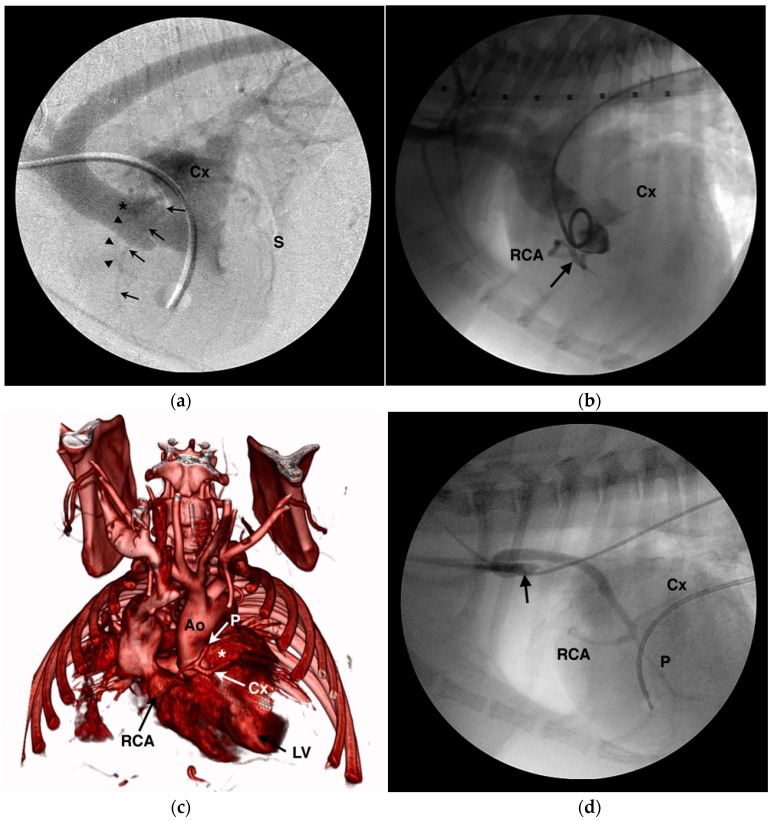
Angiograms of unique coronary artery anomalies in 4 dogs. (**a**) Levophase from a digitally-subtracted right ventriculogram in an English bulldog with pulmonary valve stenosis showing a single left coronary ostium (*) that gives a short left main coronary artery before branching into the paraconal interventricular (arrowheads), circumflex (Cx), and subsinuosal interventricular (S) branches. There is no right coronary ostium and the right coronary artery (arrows) can be seen arising from either the left main or circumflex branch. (**b**) Aortic root angiogram from an English bulldog with a single right coronary ostium that gives the right coronary artery (RCA) as well as a short left main coronary artery (arrow) that passes between the aortic root and right ventricular outflow tract before giving off the circumflex branch (Cx) and small septal or paraconal branches. (**c**) 3-dimensional, volume-rendered reformat of a computed tomography angiogram from an English bulldog with a single right coronary ostium from which arises the right coronary artery (RCA), absence of the left coronary artery, a prepulmonary course for the paraconal interventricular branch (P), and an interarterial course for the circumflex branch (Cx). A portion of the right ventricular outflow tract has been digitally removed to show the left coronary branches encircling the pulmonary trunk (*). LV = left ventricle, Ao = aorta. (**d**) Selective arteriogram from a mixed breed dog with tetralogy of Fallot whose entire coronary circulation arises off the brachiocephalic trunk (arrow), traverses lateral to the ascending aorta, and then trifurcates caudal to the aortic root into the right coronary artery (RCA) and the paraconal interventricular (P), circumflex (Cx), and subsinuosal interventricular branches.

Four canine examples of CAAs with anomalous aortic origin not previously described in the veterinary literature are shown in Figure 5. These include an English bulldog with single left coronary ostium and a retroaortic RCA (Figure 5A), an English bulldog with single right coronary ostium and transseptal LCA (Figure 5B), an English bulldog with single right coronary ostium and absent LCA in whom the paraconal interventricular branch takes a prepulmonary course while the circumflex branch takes an interarterial course (Figure 5C), and a mixed-breed dog that had tetralogy of Fallot with anomalous origin of all coronary branches from the brachiocephalic trunk (Figure 5D).

An area of debate surrounds the association between the prepulmonary arterial course and concurrent PS in bracycephalic dogs. Buchanan postulated that the prepulmonary course of the anomalous LCA caused subvalvar PS in these dogs [30]. However, the first reported case of this CAA in a dog was reported from a mixed breed dog with no evidence of pulmonary obstruction [29]. The author has also observed cases of English bulldogs with a single right coronary ostium and a prepulmonary LCA in which the right ventricular outflow tract and pulmonary valve are normal [65]. Humans with a CAA that takes a prepulmonary course do not have concurrent PS [4]. If coronary arteries, even when anomalous in course, are composed of normal cells and undergo normal growth patterns, it seems illogical that their development in a prepulmonary position would be disproportionate to the growth of the right ventricular outflow tract and lead to constriction. As the heart enlarges with growth, so to should the length of the anomalous artery and constriction of the pulmonary annulus or subpulmonary outflow tract would not develop. Given the paucity of coronary arteriograms performed in normal dogs, it may be that the prepulmonary course of a CAA is not causative but is unrelated to PS, being searched for only once PS is diagnosed. The high prevalence of PS in brachycephalic breeds [66], coupled with the apparently high prevalence of CAA in these breeds, may make these co-morbidities likely to be found in combination, even if they are unrelated. The dog whose computed tomography (CT) scan is shown in Figure 5C had no evidence of pulmonary valve stenosis even though a single right coronary ostium with a prepulmonary paraconal interventricular branch was present—indicating that these anomalies can be present distinct from the clinical scenario of PS with which they are more commonly associated. Conversely, the cranial subvalvar filling defect in the right ventricular outflow tract often seen in PS bulldogs that corresponds to the course of the prepulmonary artery does appear to contribute to the severity of obstruction in many of these patients [6,30]. In the last 33 English or French bulldogs with PS seen by the author, nine had a CAA confirmed by CT or angiography, 13 had normal coronary arterial anatomy confirmed by CT or angiography, and 11 did not undergo advanced testing leaving their coronary anatomy uncertain. Comprehensive data on non-brachycephalic breeds is less available, but in the author’s experience CAA are exceedingly rare in other breeds with PS. Further understanding of the pathogenesis and relationship of CAA to PS in dogs is needed.

**Figure 6:**
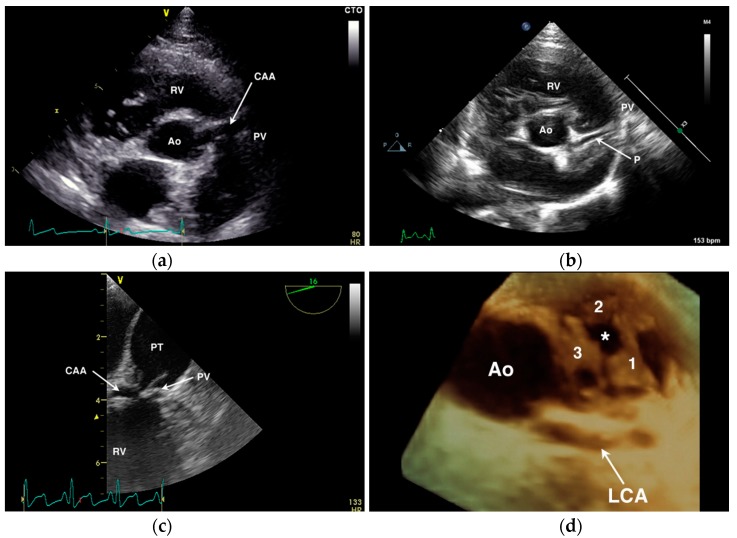
Echocardiographic examples of anomalous coronary artery origin or course. (**a**) Transthoracic short-axis image of the aortic root (Ao) showing a large single right coronary ostium with an anomalous coronary artery (CAA) traversing over the pulmonary valve (PV) annulus. RV = right ventricle. (**b**) Transthoracic short-axis image of the aortic root (Ao) from a dog with normal coronary anatomy demonstrating a normal paraconal interventricular branch (P) that appears to cross cranial to the PV annulus. (**c**) Transesophageal long-axis image of the RV outflow tract of a dog with a CAA seen crossing the PV at the base of the pulmonary trunk (PT). (**d**) Three-dimensional transesophageal image of the Ao from a dog with single right coronary ostium and a prepulmonary left coronary artery (LCA) showing the position of the LCA encircling the three PV leaflets (labeled 1 through 3) and the stenotic PV orifice (*).

**Figure 7:**
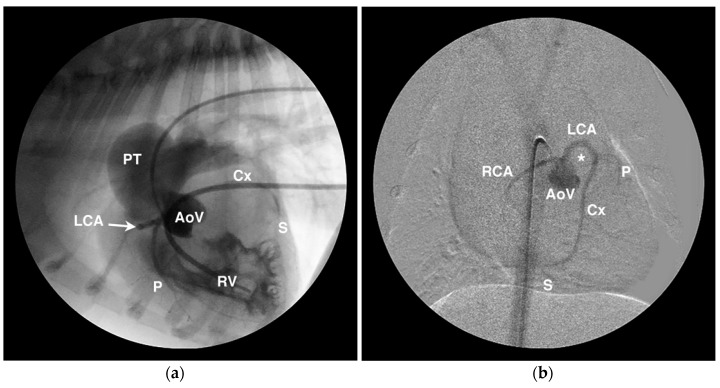
Angiographic examples of anomalous coronary artery origin and course. (**a**) Lateral angiogram from a French bulldog showing simultaneous aortic root (AoV) and right ventricular (RV) injections that demonstrate RV hypertrophy, post-stenotic dilation of the pulmonary trunk (PT), and a single right coronary ostium from which arises the left coronary artery (LCA) that encircles the RV outflow tract before branching into the paraconal interventricular (P), circumflex (Cx), and subsinuosal (S) branches. (**b**) Digitally-subtracted angiogram in a ventrodorsal oblique projection from the same dog as in panel (**a**) showing similar anatomy and highlighting the prepulmonary course of the LCA around the RV outflow tract which occupies the position highlighted by the *.

**Figure 8:**
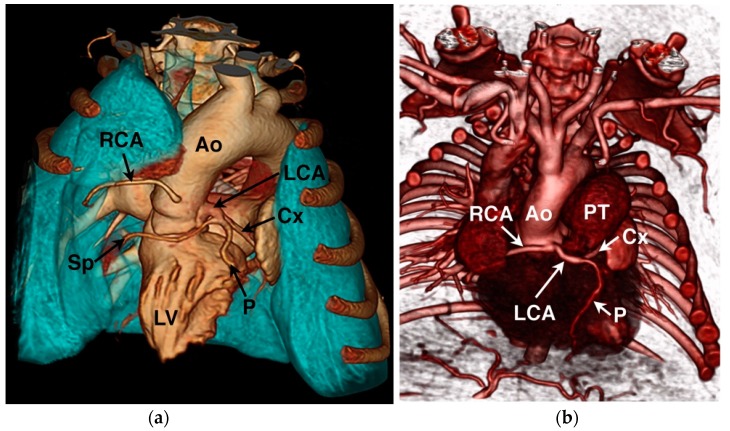
Computed tomography angiography examples of the coronary circulation from two different English bulldogs. (**a**) 3-dimensional volume-rendered reformat showing normal coronary artery anatomy. Ao = aorta, Cx = circumflex branch, LCA = left coronary artery, LV = left ventricle, P = paraconal interventricular branch, RCA = right coronary artery, and Sp = septal branch. (**b**) 3-dimensional volume-rendered reformat showing a single right coronary ostium with prepulmonary left coronary artery encircling the pulmonary valve annulus in a dog with concurrent pulmonary valve stenosis. PT = pulmonary trunk.

Diagnostic testing for anomalous coronary artery origin or course can include echocardiography (Figure 6), selective angiography (Figure 7), CT (Figure 8), and magnetic resonance imaging (MRI). In human medicine, CAA of minor clinical significance are most often diagnosed incidentally during coronary angiography [4]. When there is a suspicion of CAA, diagnostic testing in humans begins with a detailed echocardiographic evaluation, suspicious findings are confirmed by CT or MRI, and intravascular ultrasound is employed during interventional treatment to characterize the severity of ostial or luminal stenosis [4,60]. Notably, echocardiographic evaluation is not considered definitive, particularly in large persons with suboptimal imaging windows (comparable to the English bulldog) [60]. In the author’s experience in animals, transthoracic echocardiography can be suggestive of a single coronary ostium but is not definitive. Transesophageal echocardiography is more capable of determining the origin and course of the coronary vessels than transthoracic imaging [32], but is not as comprehensive as CT or MRI that allow for volume-rendered 3-dimensional reformatting of the heart (Figure 8) and a thorough evaluation of the major epicardial coronary arteries—both origin and course [34,65]. Selective coronary angiography is often diagnostic in animals, particularly when performed in two or more orthogonal planes, but is more invasive and does not inform the spatial relationship of the CAA to surrounding heart structures as can be obtained with volume-rendered reformats of CT or MRI scans.

Coronary artery stent-angioplasty, surgical unroofing of an intramural coronary segment, or osteoplasty to create a new opening in the appropriate valvar sinus are treatment strategies in humans with single coronary ostium in whom myocardial ischemia is present or perceived to be a risk [4].

The appearance of a vessel (parallel double-line echoes) overlying the right ventricular outflow tract on transthoracic echocardiography should be interpreted cautiously. While this finding may suggest a CAA with prepulmonary course (Figure 6A), in the author’s opinion it is not definitive. It is possible to create an image where normal coronary vessels appear to overlie or cross the right ventricular outflow tract—both the accessory branch of the RCA as well as the paraconal interventricular branch of the LCA (Figure 6B) can lie in close proximity to the pulmonary valve and right ventricular outflow tract confusing the diagnosis of a prepulmonary coronary arterial course. With transesophageal echocardiography (Figure 6C), it is possible to more thoroughly interrogate the aortic valvar sinuses for presence/absence of coronary ostia and to follow their proximal course. High take-off ostia or suboptimal imaging planes may still limit conclusive imaging with this modality and determining the course of the coronary vessels beyond their proximal origin can be challenging. Considerable experience is necessary to correctly diagnose coronary anatomy by 2-dimensional echocardiography; 3-dimensional imaging in the author’s experience provides a better overview and a more complete ‘en face’ view of the pulmonary annulus and relationship of a prepulmonary arterial course (Figure 6D). In human medicine, 2-dimensional transesophageal echocardiographic diagnosis of the precise CAA present showed poor agreement with surgical findings and marked differences of interpretation when reviewed by a core laboratory [27]; an evaluation of the potential improvement afforded by 3D transesophageal echocardiographic imaging is not available, to the author’s knowledge. As the therapeutic options and the prognostic significance of correctly identifying a CAA in the setting of PS differ widely from a mistaken diagnosis, the author advises caution in making a definitive diagnosis by 2-dimensional echocardiography and still recommends angiography or cross-sectional imaging in most cases.

### 4.2. Anomalous Origin from the Pulmonary Trunk

Anomalous origin of a coronary artery from the pulmonary trunk results in myocardial steal and a left-to-right shunt as the low pulmonary vascular resistance drives blood flow away from the myocardium and to the pulmonary circulation. Reports in animals are rare, being described in a dog [38] and several bovine cases [47,48,49]. Re-implantation to the aorta is the preferred treatment strategy in humans, though ligation of the LCA at the pulmonary valvar sinus may be a feasible treatment strategy in the dog to resolve shunt flow if sufficient collateral flow to the myocardium is provided through the RCA.

## 5. Coronary Arterial Fistulae

**Figure 9:**
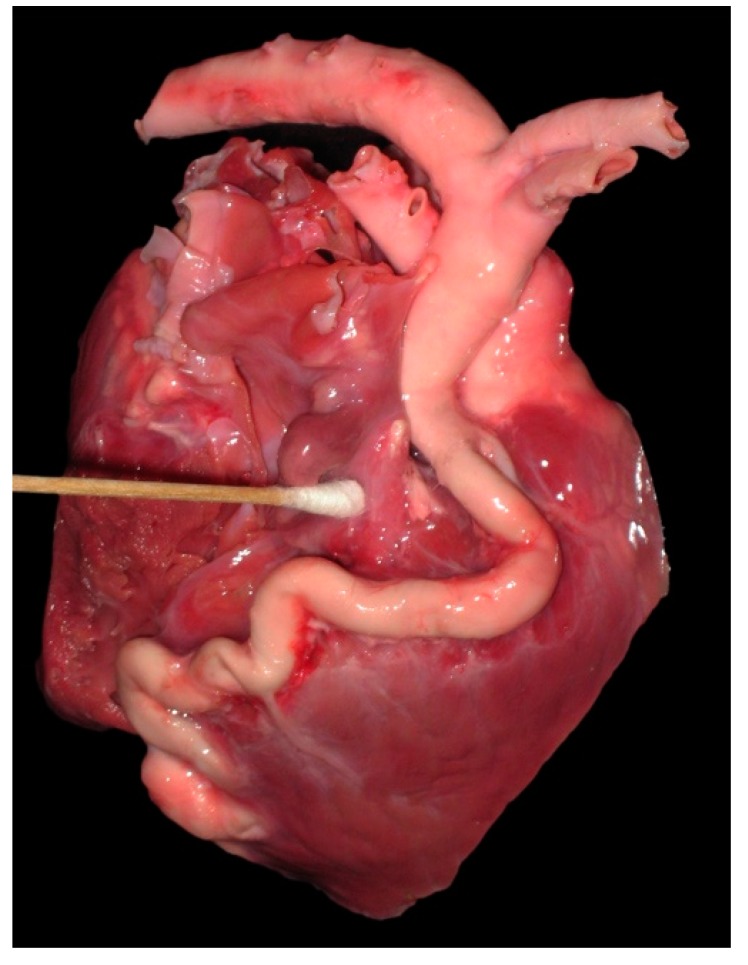
Photograph of the heart from a 6-day-old llama cria showing a severely dilated and tortuous right coronary artery fistula, which drained to the proximal right ventricle.

Fistulous connections between the coronary arterial system and the cardiac chambers (coronary cameral fistula), the pulmonary circulation, or the vena cavae are occasionally observed in animals. This manuscript specifically refers to congenital CAA, though acquired aortocardiac fistula may present similarly and are most often reported in the horse [67]. Termed coronary cameral fistulae when they drain to a cardiac chamber [68], direct connections of the coronary arteries in humans arise from the RCA in 52%, the paraconal interventricular branch in 30%, and the circumflex branch in 18% of cases [69]. Clinical signs and cardiac remodeling vary by the size of the fistula (amount of flow) and the chambers involved with left heart enlargement expected in most cases and right heart enlargement noted when the fistula drains to the right atrium or right ventricle [68,69]. These lesions may be isolated (Figure 9), but can also be observed in combination with ventricular hypoplasia such as pulmonary atresia with intact ventricular septum when the suprasystemic pressure in the right ventricle forces blood into primitive vascular channels that connect with the coronary circulation [70].

**Figure 10:**
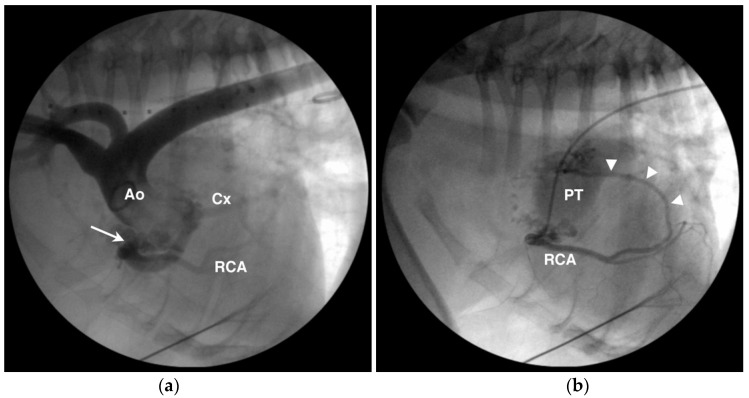
Angiographic images from an English bulldog with single right coronary ostium, aneurysmal dilation of the prepulmonary left coronary artery and circumflex branch (Cx), and coronary to pulmonary arterial fistulae/malformation. (**a**) The aortic root (Ao) injection shows the single right coronary ostium (arrow), aneurysmal dilation of the Cx, and a more normal diameter right coronary artery (RCA). (**b**) Selective right coronary angiogram from the same dog as in (**a**) showing one of several fistulous connections (arrowheads) from the RCA to the pulmonary trunk (PT).

Coronary to pulmonary artery fistulae to the pulmonary circulation are occasionally observed in animals, most often in the author’s experience as part of a larger thoracic arteriovenous malformation [71], though isolated coronary to pulmonary arterial connections also occur (Figure 10). 

## 6. Coronary Artery Aneurysm

Coronary artery aneuryms, also termed coronary artery ectasia, may be congenital or acquired. Reports in dogs have been in conjunction with subaortic stenosis [39], and fistulous vessels in published reports are often aneurysmal similar to that seen in Figure 9. An example of an aneurysmal RCA is provided in Figure 10 from an English bulldog with a single right coronary ostium, prepulmonary LCA, and fistulous connections to the pulmonary arterial circulation consistent with an arteriovenous malformation. The clinical significance of coronary artery aneurysms is uncertain and therapeutic intervention has not been reported in an animal. In humans, coronary artery aneurysms are typically acquired secondary to atherosclerotic, infectious, or connective tissue disease processes [72]. Therapy in people concentrates on limiting thromboembolic risk with antiplatelet and anticoagulant medications, implantation of covered stents, or surgical excision [72].

## 7. Myocardial Bridging of Epicardial Coronary Arteries

**Figure 11:**
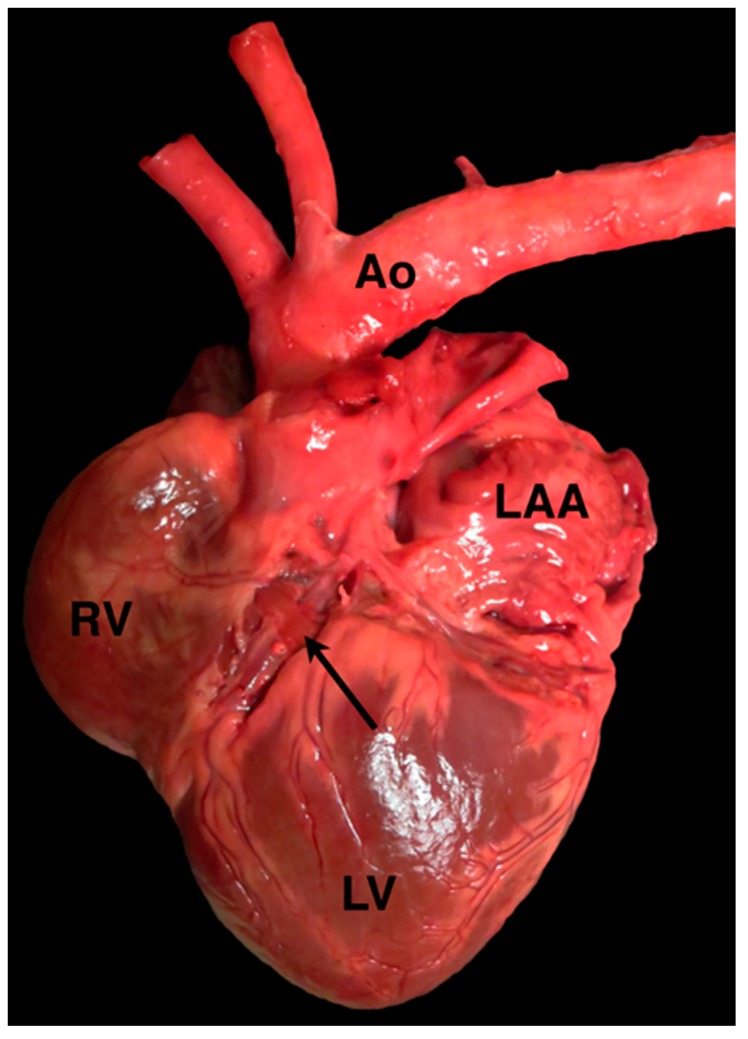
A photograph of a myocardial bridge (arrow) covering a portion of the left paraconal interventricular branch of the left coronary artery in a dog. Ao = aorta, LAA = left auricular appendage, LV = left ventricle, RV = right ventricle.

Myocardial bridging is an anatomical anomaly seen in human [73], canine [42], and porcine [74] hearts where the epicardial coronary arteries tunnel below a section of myocardium. Veterinary studies[42,74] suggest prevalence of up to 30% in dogs and near 50% in swine, which are comparable to human reports [73]. The clinical significance of this anomaly is uncertain as the majority of coronary arterial flow occurs in diastole, while constriction of the coronary artery by a myocardial bridge is predominately a systolic event. However, the size of the tunneled segment, concurrent left ventricular hypertrophy, tachycardia and a shortened diastolic perfusion time, and coronary spasm associated with the myocardial bridge all may play a role in impairing myocardial perfusion and result in clinical signs in humans [73]. Whether similar pathophysiology occurs in animals is presently unknown. An example of a myocardial bridge incidentally detected on post mortem evaluation is shown in Figure 11.

## 8. Conclusions

Anomalies of the coronary artery circulation have been observed in nearly all domesticated species both in isolation and in association with concurrent congenital heart disease. Understanding the anatomy, terminology, and clinical implications of these CAA are important both in the treatment of veterinary patients as well as serving as potential models for understanding CAA in humans. As the diagnostic capabilities available to veterinary cardiologists improve, it is likely that additional anomalies of the coronary arterial circulation will be found.

## Figures and Tables

**Table 1:** Categorization of congenital coronary artery anomalies; modified Dodge-Khatami et al. [57].

**Major Coronary Artery Anomalies**
Anomalous origin from the pulmonary artery
Left coronary artery from PA ^a,b^
Right coronary artery from PA
Both coronary arteries from PA
Coronary cameral fistula
Draining to RV ^b,c^
Draining to RA
Draining to LV ^b^
Draining to LA
Coronary to pulmonary arterial shunt ^a,b^
Congenital atresia of the LCA
**Minor Coronary Artery Anomalies**
Anomalous origin from the aorta
Origin of LCA from right aortic sinus ^a,b,d^
Origin of RCA from left aortic sinus ^a,e^
Origin of paraconal branch from right aortic sinus ^a^
Origin of paraconal branch from RCA
Origin of circumflex branch from right aortic sinus ^a^
Origin of circumflex branch from RCA
Single coronary artery
Single left coronary artery
Single right coronary artery
Inverted coronary arteries
Other anomalous aortic origin ^a^
Anomalous epicardial course
Prepulmonary course ^a,b,d,^*
Interarterial course ^a,b,d^
Transseptal course ^a^
Retroaortic course ^a^
Duplication of a coronary artery
Duplication of paraconal branch
Duplication of circumflex branch
Duplication of RCA
Multiple ostia within left aortic sinus ^a^
Multiple ostia within right aortic sinus ^a^
High take-off coronary artery ^a^
Coronary artery aneurysm ^a^
Myocardial bridging of an epicardial coronary artery ^a,e^

^a^ = canine examples are reported, presented in this paper, or have been observed by the author; ^b^ = bovine examples are reported, presented in this paper, or have been observed by the author; ^c^ = camelid examples are reported, presented in this paper, or have been observed by the author; ^d^ = equine examples are reported; ^e^ = porcine examples are reported; * this anomaly has major clinical significance in the setting of concurrent pulmonary valve stenosis.
